# Regional hyperthermia of the abdomen, a pilot study towards the treatment of peritoneal carcinomatosis

**DOI:** 10.1186/s13014-015-0451-3

**Published:** 2015-07-30

**Authors:** Marcus Beck, Pirus Ghadjar, Mirko Weihrauch, Susen Burock, Volker Budach, Jacek Nadobny, Jalid Sehouli, Peter Wust

**Affiliations:** Department of Radiation Oncology, Charité Universitätsmedizin Berlin, Augustenburger Platz 1, 13353 Berlin, Germany; Charité Comprehensive Cancer Center, Charité Universitätsmedizin Berlin, Berlin, Germany; Department of Gynecology, Charité Universitätsmedizin Berlin, Berlin, Germany

**Keywords:** Hyperthermia, Heat, Cancer, Peritoneal carcinosis, Heatability

## Abstract

**Background:**

Peritoneal carcinomatosis occurs in different cancer subtypes and is associated with a dismal prognosis. Some doubts remain whether the whole abdomen can be treated by regional hyperthermia, therefore we analyzed feasibility conducting a pilot study.

**Methods:**

A simulation of the abdominopelvic heat distribution in 11 patients with peritoneal carcinomatosis was done using the HyperPlan software and the SIGMA-60 and SIGMA-Eye applicators. Tissue-specific region-related electrical and thermal parameters were used to solve the Maxwell’s equations and the bioheat-transfer equation. Three-dimensional specific absorption rate (SAR) distributions and, additionally, estimated region-related perfusion rates were used to solve the bioheat-transfer equation. The predicted SAR and temperature distributions were compared with minimally invasive measurements in pelvic reference points.

**Results:**

In 11 patients (7 of them treated in the SIGMA-60 and 4 in the SIGMA-Eye applicator) the measured treatment variables (SAR, temperatures in the pelvic reference points) indicated that the heated volumes were higher for the SIGMA-Eye applicator. The mean computed abdominal SARs were less for the SIGMA-Eye (33 versus 44 W/kg). Nevertheless, the temperature distributions in the abdomen (peritoneal cavity) were more homogeneous in the SIGMA-Eye applicator as compared to the SIGMA-60 as indicated by higher values of T_90_ (mean 40.2 versus 38.2 °C) and T_50_ (mean 41.1 versus 40.2 °C), while the maximum temperatures were similar (in the range 41 to 43 °C). Even though the mean abdominal SAR was lower in the SIGMA-Eye, the heat distribution covered a larger volume of the abdomen (in particular the upper abdomen).

For the SIGMA-60 applicator the achieved T_90_ appeared to be limited between 41 and 42 °C, for the SIGMA Eye applicator more effective T_90_ in the range 42 to 43 °C were obtained.

**Conclusion:**

Our results suggest that an adequate heating of the abdomen and therefore abdominal regional hyperthermia in PC patients appears feasible. The SIGMA-Eye applicator appears to be superior compared to the SIGMA-60 applicator for abdominal hyperthermia.

## Introduction

Peritoneal carcinomatosis (PC) occurs in different cancer subtypes and is associated with a dismal prognosis. In non-gynecologic malignancies such as colorectal, gastric, pancreatic cancer as well as in peritoneal mesothelioma, pseudomyxoma peritonei and primary peritoneal carcinoma a median overall survival (OS) of 3,1 months was reported [1, 2].

On the other hand, ovarian cancer is the most frequently type of gynecologic cancer accompanied by PC. In a single center analysis of 214 patients with primary ovarian cancer the initial surgery detected a peritoneal manifestation in 76 % [3]. The 5 year OS in advanced stage ovarian cancer (Stage III/IV) is approximately only 25 % [4].

During the last decades several efforts have been made to improve the therapy of PC. Besides intravenous chemotherapy and cytoreductive surgery (CRS), the intraperitoneal chemotherapy and lastly CRS followed by hyperthermic intraperitoneal chemotherapy (HIPEC) were implemented. CRS and HIPEC were claimed to be beneficial for patients with pseudomyxoma peritonei, peritoneal mesothelioma and colorectal cancer [5, 6]. Less is known regarding the role of CRS and HIPEC for PC in gastric cancer and for ovarian cancer [7–11]. As a general limitation CRS is associated with notable morbidity, mortality and a restriction of suitable patients [7–11]. Therefore abdominal regional hyperthermia (RHT) combined with intravenous chemotherapy appears to be an important non-invasive and well tolerated option in the treatment of PC [12]. In two phase I/II studies at our center we demonstrated that this approach was associated with encouraging OS rates and low RHT associated toxicities in patients suffering from PC of colorectal and ovarian cancers [13, 14]. However, the beneficial effect of RHT for the treatment of PC has never been demonstrated in a randomized trial. Additionally, there were some doubts whether RHT, using commercially available applicators (such as the SIGMA-60 and SIGMA-Eye), could actually lead to an appropriate heat distribution covering the whole abdomen (including the peritoneal cavity). To facilitate the future use of RHT for the treatment of PC as well as the need for innovative randomized trials incorporating RHT we performed a planning study to evaluate whether RHT of the abdomen using SIGMA-applicators is feasible.

## Methods

We generated patient models for a group of 11 treated patients with PC. These 11 patients suffered from PC originating from different cancer subtypes and were randomly selected from a larger cohort which underwent RHT in the SIGMA-60 applicator or SIGMA-Eye applicator within a phase I/II trial, as previously reported [13, 14]. On computed tomography (CT) scans we segmented the target volume (abdominal cavitiy) and surrounding organs and structures like liver, kidneys, bones, muscles, fatty tissue, vagina and bladder. Than specific patient models with individual tetrahedron grids were generated and the finite elements method (FEM) was used for the model calculations. The specific absorption rate (SAR)-distributions (W/kg) were calculated using the treatment planning system HyperPlan as previously described [15, 16]. For the simulations we used assumed electrical, thermal and physiological parameters of the tissues according to the values previously reported. See [17–19] and Table 1. The parameters for permittivity and conductivity showed no relevant frequence dependend variety in our measurement (70-100 MHz) and so the value for 90 MHz was used. These parameters in conjunction with predicted SAR distributions were used to solve the bioheat-transfer equation. Applicator models for the SIGMA-60 applicator (8 dipole antennas arranged in pairs and connected to 4 amplifier channels) and SIGMA-Eye applicator (24 dipole antennas arranged in pairs and connected to 12 amplifier channels) were available. The hyperthermia planning system HyperPlan has been tested and validated for both applicator models [15, 16].

**Table 1:** Electrical parameters for computation of the power deposition patterns and perfusion parameters (90 MHz)

Tissue	*Ɛ*_*r*_	σ(S/m)	Perfusion under RHT (ml/100g/min)
Muscle	80	0.8	30
Fat	10	0.04	20
Abdomen	36	0.55	20
Liver	78	0.6	100
Kidneys	83	1	400
Rectum	60	0.7	5
Perirectal tissue	23	0.3	20
Vagina	80	0.8	5
Bladder	80	0.6	5

*Ɛ*_*r*_ relative permittivity_,_ *σ(S/m)* conductivity

**Fig. 1:**
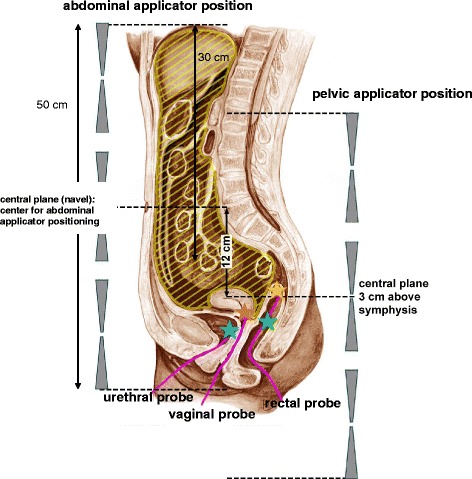
Arrangement of the annular-phased-array applicator, centered on the navel, for abdominal heating and centered 3 cm above symphysis for pelvic heating. Sometimes the applicator position for SIGMA-60 differed from central plane as shown in Table 2. The reference points in endoluminal catheters are also depicted (marked with colored stars)

The SAR and temperature distributions predicted via HyperPlan can be compared with measurements in single pelvic reference points in the rectum, vagina and bladder (marked with star symbols in Fig. 1) by using the point probe software tool of HyperPlan, which enables a measurement of SAR or temperature of every specified point. The measurement of SAR was performed using the temperature decline in the time-temperature curve after turning of the power after treatment.

Perfusion and its regulation during heat exposure is a crucial characteristic of the tissues in order to compute the temperature distribution. Liver and kidneys are the highest perfused organs. The perfusion of the abdomen was estimated as 20 ml/100g/min by assuming a mixture of the intestine (basal perfusion of 40-50 ml/100g/min) and surrounding low perfused connective tissue. The gastrointestinal tract has a large potential to increase perfusion during digestion (up to 6 fold) and has also (as other organs) a self-protection ability against temperature elevation. However, the thermoregulation in the visceral organs is less pronounced than in other tissues (e.g. skin) because the temperature in the portal vein might be as high as 40 °C under physiological conditions (caused by chemical processes during digestion). Therefore, a higher tolerance against temperature increase might be assumed in visceral organs. Furthermore, the intestinal perfusion might even be depressed under somatic stress such as regional hyperthermia. Therefore, 20 ml/100g/min is a reasonable approach to describe the peritoneal cavity [19]. The maximum temperature in the organs such as intestine, rectum and bladder was set to 43 °C. The perfusion in other normal tissues such as muscle, fat or perirectal tissue is under hyperthermic conditions higher than the basal perfusion (Table 1).

In a first step, we reviewed for every patient, whether the measurements in the reference points (both SAR and temperature) could be correctly reproduced by the corresponding patient-specific plan (using HyperPlan). The computations are based on the patient anatomy (as basis for a patient model), the treatment variables (frequency, total power, phase amplitudes) and the tissue parameters according to Table 1. We started with the applicator position described in the treatment protocol, but for matching the plan to the measured data a slight variation of the positions of the applicator and the point probes was admitted. Variations of the positions in relation to the navel central plane (Fig. 1) were listed in Table 2. Due to patient discomfort in some cases it was necessary to shift the SIGMA-60 applicator a few cm distal the navel as listed in Table 2.

**Table 2:** Analysis of the SAR and temperature distribution in the abdomen

SIGMA-60	SAR _50_ (abdomen) (W/kg)	T _90_ (°C)	T _50_ (°C)	T _max_ (°C)	SIGMA-Eye	SAR _50_ (abdomen) (W/kg)	T _90_ (°C)	T _50_ (°C)	T _max_ (°C)
70 MHz, 700 W, −2 cm	45	38,3	40,1	41,8	100 MHz, 1500 W	35	40,7	41,6	43,0
90 MHz, 600 W, −3 cm	42	38,3	40,6	42,7	100 MHz, 1600 W	36	39,9	41	42
90 MHz, 500 W	42	38,1	39,5	41,2	100 MHz, 1500 W	20	39,1	39,8	41,2
90 MHz, 600 W	43	38,2	40,7	42,2	100 MHz, 1500 W	40	41	41,9	42,8
90 MHz, 500 W, −3 cm	52	39,1	41	43,7	Mean value	33	40,2	41,1	42,2
70 MHz, 500 W, −4 cm	32	38	39,5	41,3					
90 MHz, 550 W	40	37,6	39,8	42,2					
Mean value	42	38,2	40,2	42,2					

The applicator position is centered on the central plane (navel) as shown in Fig. 1. Distal aberrations (in cm) of applicator position to central position (required for a few patients treated with Sigma-60) are listed in the left column

*SAR* specific absorption rate

In a second step, we evaluated the SAR and temperature distributions from HyperPlan in the abdomen. We used the described individual patient models, which were assumed as validated after successful matching in the first step.

In a third step, we varied (in particular increased) the total power in the available patient models to estimate the limitations of abdominal heating (with ring applicators) under favorable conditions. The total power was restricted, if a temperature threshold in any tissue was exceeded. Because the total power is increased in steps of 100 W, such a maximum permissible temperature might be slightly passed in the simulation studies and the generated temperature plots. We assume a self-protection of all healthy tissues by increasing the perfusion, if we go only slightly beyond the threshold.

This analysis was conducted in accordance with the regulations of the local Ethics Committee of the Charité Universitätsmedizin Berlin.

## Results

In 11 treated patients (7 of them in the SIGMA-60 and 4 in the SIGMA-Eye applicator) particular treatment variables (SAR, temperatures in the reference points) were determined. We selected a typical heating session for every patient. The average values for total power applied, absolute SAR and steady-state temperatures in the reference points for all patients on each applicator were created: For the SIGMA-60 applicator 500-700W, SAR(vagina) 22 W/kg, SAR(rectum) 16 W/kg, T_vagina_ 40,8 °C and T_rectum_ 40,3 °C. For the SIGMA-Eye applicator 1400-1600W, SAR(vagina) 11 W/kg, SAR(rectum) 2 W/kg, T_vagina_ 40 °C and T_rectum_ 39,4 °C. In all treatments the phase delays were 0 and amplitudes were equally weighted. No pattern optimization was performed, because we assumed that the balanced phase and amplitude control is near the optimum for the purpose of abdominal heating.

The measured values indicate that the heated volumes are higher for the SIGMA-Eye applicator, which needs therefore higher total power. Nevertheless, the mean SAR and temperatures in the pelvis are lower in the SIGMA Eye applicator in comparison to the SIGMA-60 applicator. However, the considerably higher total (amplifier) power required at the 12 amplifiers of the SIGMA-Eye is mainly due to a lower efficiency, which is nearly half of the SIGMA 60´s efficiency. The lower efficiency is caused by losses in the matching networks (of short antennas) and feeding cables. Furthermore, this SIGMA-Eye applicator was used in a hybrid system where additional loss of efficiency is caused by specifics of the electro-technical separation of MRI and applicator [13]. All these attributes had been included into the applicator models used in HyperPlan. Note that the power levels given in this study for the SIGMA-Eye applicator are different from power levels in a SIGMA-Eye applicator outside the MRI.

**Fig. 2:**
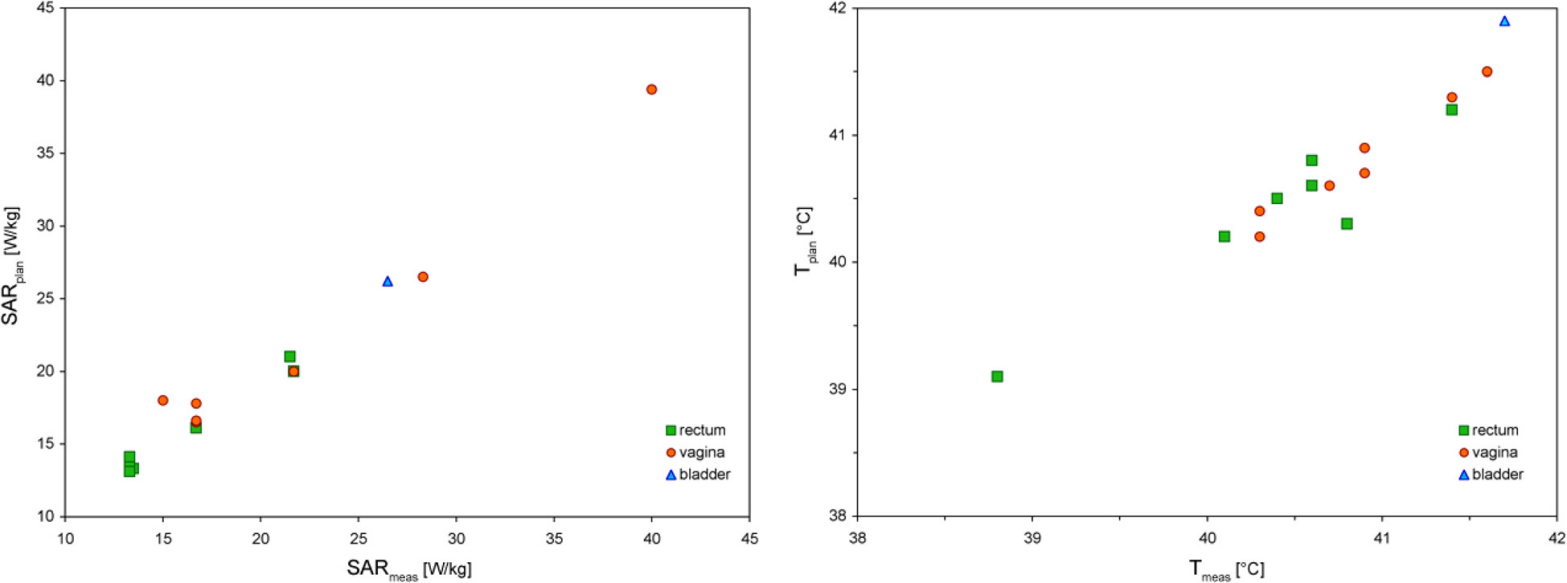
Correlation of measured SAR in the reference points and calculated values in HyperPlan after a matching process (see *left*). Correlation of measured and calculated temperatures in the same reference points under the same conditions (see *right*). *r* = 0.997 for SAR and *r* = 0.90 for temperatures

We compared the measured temperatures and SAR in the reference points with the values in the corresponding patient-specific plans and performed a matching of all measurements simultaneously by adapting the applicator and point probe positions (see Methods). We found in all patients already a satisfactory agreement in the first approximation, but typically further improvement was possible by a straight forward search in an unambiguous direction of the parameter space. Finally, we achieved a fair correlation as depicted in Fig. 2 with a correlation coefficient of *r* = 0.997 for SAR and *r* = 0.90 for temperatures, respectively.

After completion of matching procedure we computed the SAR and the temperature distributions in the whole volume (pelvis and abdomen), using the verified patient models and the refined positions of the central plane (longitudinal shift from the belly button or navel). We achieved median SAR and index temperatures in these 11 patient models as summarized in Table 2.

We noted characteristic differences between both applicators according to Table 2. The mean SAR were less for the SIGMA-Eye (33 versus 44 W/kg). This was mainly due to the lower SAR in the pelvic region by using the SIGMA-Eye (see also Table 2). Nevertheless, the temperature distributions in the abdomen (peritoneal cavity) were more homogeneous in the SIGMA-Eye applicator, which was indicated by higher T_90_ (40.2 versus 38.2 °C on average) and T_50_ (41.1 versus 40.2 °C on average), while the maximum temperatures were similar (in the range 41 to 43 °C). Even though the mean SAR was lower in the SIGMA-Eye, the distribution covered a larger volume of the abdomen (in particular the upper abdomen). Therefore, the T_90_ for the SIGMA-Eye were above 40 °C on average, which might be sufficient for some sensitizing effect in conjunction with chemotherapy (and radiotherapy).

**Fig. 3:**
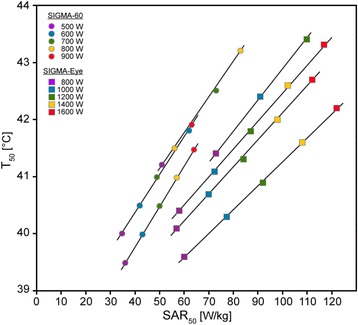
Simulated characteristic lines in a SAR/temperature diagram for both applicators and the available patient models by variation of total power until a threshold temperature is exceeded. Classification in easy (lines shifted to the *left*) - and difficult-to-heat (lines shifted to the *right*) patients. As the curves were very similar for patients treated in the Sigma-60 applicator we show only 3 curves for this applicator (the remaining curves would lie in the interspace between the depicted curves) to enhance the clarity of the figure

We proceeded with the simulation study by further increase of total power starting from the actual matched treatments with the SIGMA-60 or the SIGMA-Eye applicator. The SAR_50_ and T_90_ in the peritoneum belonging to a particular power level and applicator are determined plotted in a two-dimensional coordinate system (Fig. 3). The plotted points rearrange in characteristic lines corresponding to certain patients and applicators.

The total power was always turned off after a critical temperature was exceeded (43 °C in the organs). Under these conditions we could differentiate between easy-to-heat (lines shifted to the left) and difficult-to-heat patients (lines shifted to the right). For the SIGMA-60 applicator the achieved T_90_ appeared limited between 41 and 42 °C, and only exceptionally T_90_ reached 43 °C. For the SIGMA Eye applicator we had to apply higher total power levels and obtained a more effective T_90_ in the range of 42 to 43 °C. Even for the most difficult-to-heat patient we finally achieved a T_90_ of 42 °C without exceeding a temperature threshold. Evidently, we could heat a higher volume of the abdomen with more homogeneity and a better covering by use of the SIGMA Eye applicator.

**Fig. 4:**
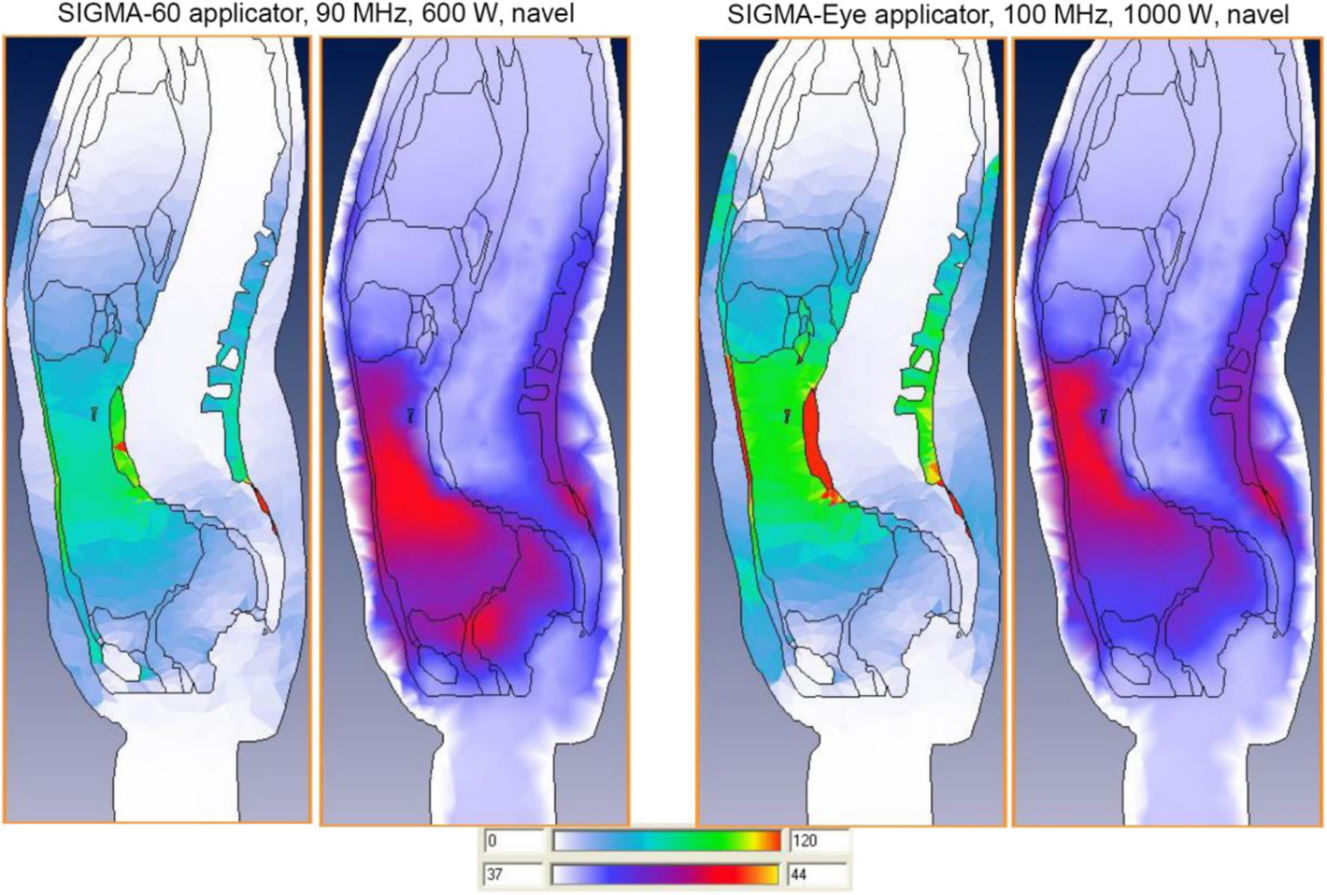
Comparison of typical SAR and temperature patterns gained with the SIGMA-60 applicator (*left*) and the SIGMA-Eye applicator (*right*). In the SIGMA-60 the pelvic region is more effectively heated, while the upper abdomen is better covered in the SIGMA-Eye applicator. The dorsal temperature hotspot represents a local effect on the border of bone and fatty tissue with no consequence for nearby structures, particularly there is no critical temperature enhancement in the bony shielded spinal canal

The different patterns are illustrated by Fig. 4, which shows SAR and temperature distributions for both applicators under comparable conditions for a selected model. Both applicators were centered on the navel plane. The SIGMA-60 applicator tended to heat the pelvis more effective. However, the SIGMA-Eye applicator covered the upper abdomen better and gained after all a better T_90_ for the whole abdomen.

**Fig. 5:**
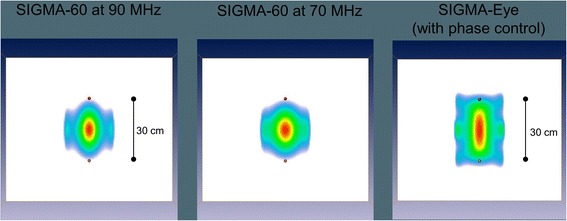
Comparison of the pattern of effective heated abdominal volume for SIGMA-60 applicator (frequencies of 90 MHz and 70 MHz) and the broadened pattern enabled by proper phase control in SIGMA-Eye applicator

**Fig. 6:**
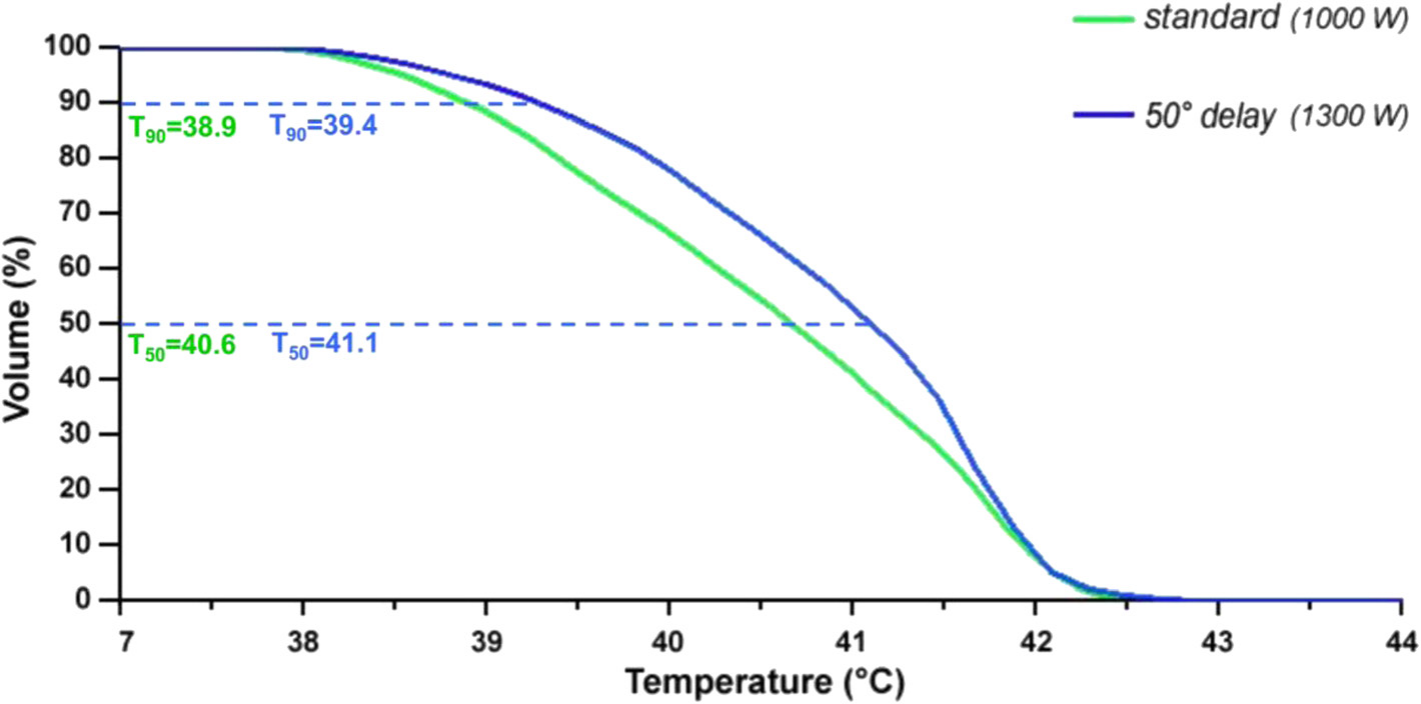
Changes of effective heated volume using SIGMA-Eye applicator with standard adjustment and under proper phase control with a delay of 50° on the outer applicator rings

The pattern of the SIGMA-Eye applicator may even be improved by proper phase control. In our simulations we could show the effect of a delay on the outer rings of the applicator resulting in a broadening of the effective heating volume and an increased SAR. A phase delay of 50° at the outer rings was performed. The opportunity of proper phase control of SIGMA-Eye in comparison to SIGMA-60 and the resulting effects in effective heating volume are depicted in Figs. 5 and 6. Note that for the larger volume an elevation of the total power is also necessary in order to ensure a superior temperature distribution with higher T_90_.

## Discussion

Our simulation study based on clinical experience and measurements suggests an appropriate abdominal heating capability using the annular-phased-array technique. Furthermore our results show the applicability of SIGMA-60 as well as SIGMA-Eye with a small advantage of SIGMA-Eye applicator in abdominal RHT.

CRS/HIPEC, with its notable treatment related toxicity and mortality rates, are objective of several ongoing and completed retrospective and prospective trials. As reported in retrospective and phase I/II trials there appears to be a benefit for patients with pseudomyxoma peritonei, peritoneal mesothelioma and colorectal cancer with PC [5, 6, 20, 21]. However, for other cancer types e.g. ovarian cancer even less supportive data for CRS/HIPEC exist. Notably, even in experienced centers CRS/HIPEC procedures were associated with toxicity rates between 0 and 40 % and mortality rates of 0–10 % and only a limited number of patients (after careful selection) are actually eligible for CRS/HIPEC. In patients with PC of non gynecologic origin grade 3–4 toxicity rates range from 29 to 40 %, whereas in ovarian cancer PC patients in 0–32 % grade 3–4 morbidity was reported. Moreover in gynecologic patients grade 3–4 hematologic morbidity of 8–31 % was observed due to application of the intraperitoneal chemotherapy [7–11]. Therefore, it appears unlikely that CRS/HIPEC will truly improve the therapeutic ratio for a large number of patients suffering from PC and thus, other preferably non-invasive and better tolerated approaches are needed to intensify the treatment of patients with PC.

Experiences with whole body hyperthermia (WBH) showed promising results in several Phase II studies treating colorectal, ovarian and other cancer subtypes. However, it is associated with a grade III/IV toxicity rate of 3.1 % in 1300 described treatments. Moreover, the rate of deaths in the first 30 days after WBH plus chemotherapy treatment was 0.8 %. Furthermore WBH requires general anaesthesia or deep analgesic sedation and is therefore also logistically demanding. Clinical experience shows that WBH is limited to 3-6 sessions because of this burden. Therefore, WBH is neither for a palliative nor for a maintenance approach suitable [22–24].

In contrast RHT has been demonstrated as effective and is associated with a very low rate of treatment related toxicities [12–14]. But it is important to consider current quality assurance guidelines for treatment planning and application [25, 26]. Moreover, with few exceptions (pace maker, severe cardiac disease, metal implants in the treatment area, acute thrombosis), RHT has less strict inclusion criteria and can potentially be applied to a larger proportion of patients suffering from PC than CRS/HIPEC and WBH with its limitations as described before. So far no randomized trial was initiated to test the combination of RHT and chemotherapy vs. chemotherapy alone. Our group is currently developing a randomized phase II trial who will address this question in patients with recurrent ovarian cancer.

There are several limitations to our study. A major problem are direct (invasive) temperature measurements in the abdomen/peritoneum. However, there are substantial practical obstacles to perform invasive measurements in these seriously sick patients. Invasive thermometry would require a laparoscopic placement or at least a radiological intervention. Both procedures ar associated with morbidity and discomfort (and even risks of complications as bleeding or infection). In particular, invasive thermometry is not suitable for routine use. On the other hand, we widely used minimally invasive temperature measurements with endoluminal catheters in rectum, vagina and/or bladder. The replacement of invasive intra-tumoral measurements by endoluminal reference measurements (Fig. 1) is a generally applied method and has been extensively discussed elsewhere [27, 28]. Here, we combined potent planning tools matched to reference measurements in order to achieve the information outlined in this paper. This might be an acceptable strategy for a first elucidation of abdominal heating. Abdominal heating has a wide spectrum of oncological indications (pancreatic cancer, ovarian cancer, colorectal cancer, gastric cancer and peritoneal carcinomatosis of different kinds), probably more than in case of pelvic heating. For a broad application of abdominal heating in the future, better specific quality parameters a strongly desirable. Noninvasive thermometry (MRI-thermometry) is the method of choice to obtain the information needed with low morbidity and clearly has much better acceptance in comparison to invasive and minimally invasive thermometry [29]. However until now, noninvasive MRI-thermometry in the abdomen is still a great challenge. Inhomogeneities and motion are great obstacles to implement noninvasive MRI-thermometry under routine conditions. For abdominal heating MRI-thermometry of the liver could be a promising concept, because it appears feasible and would provide the mean temperature of the target, i.e. abdomen. Another point of criticism might be the assumption of 20 ml/100g/min for the abdominal perfusion which has been estimated considering current physiologic knowledge and in accordance with former studies [19]. In case of incorrect perfusion parameters the temperature distribution would change. For example a higher perfusion rate would lead to lower abdominal temperatures. Again, MRI-thermometry will elucidate the behavior of abdominal perfusion under heating conditions, which is a complex and not yet completely resolved physiological issue. Despite these objections we believe that an appropriate abdominal heat distribution can be achieved by RHT and further studies with RHT in the future are warranted in patients with PC.

## Conclusion

Our results suggest an adequate heating of the abdomen and therefore abdominal regional hyperthermia in PC patients appears feasible. This may facilitate the use of RHT in future multidisciplinary trials for the treatment of PC. To ensure a qualitative regional abdominal hyperthermia the combination of a hyperthermia treatment planning and non invasive MRI-thermometry should further be developed to be a standard combination in future treatments [25]. The SIGMA-Eye applicator appears to be the optimal applicator for abdominal RHT.
